# Involvement of B cells in the development of systemic sclerosis

**DOI:** 10.3389/fimmu.2022.938785

**Published:** 2022-07-28

**Authors:** Ayumi Yoshizaki, Takemichi Fukasawa, Satoshi Ebata, Asako Yoshizaki-Ogawa, Shinichi Sato

**Affiliations:** Department of Dermatology, The University of Tokyo Graduate School of Medicine, Tokyo, Japan

**Keywords:** systemic sclerosis (scleroderma), B cell, autoimmune disease, cytokine, effector B cell, regulatory B cell

## Abstract

Systemic sclerosis (SSc) is a rare intractable systemic disease that causes fibrosis and vasculopathy against a background of autoimmune abnormalities. Although the etiology is not yet fully understood, the type of autoantibodies detected in SSc is closely associated with disease severity and prognosis, supporting that those autoimmune abnormalities play an important role in the pathogenesis of SSc. Although the direct pathogenicity of autoantibodies found in SSc is unknown, many previous studies have shown that B cells are involved in the development of SSc through a variety of functions. Furthermore, a number of clinical studies have been conducted in which B-cell depletion therapy has been tried for SSc, and many of these studies have found B-cell depletion therapy to be effective for SSc. However, the involvement of B cells in pathogenesis is complex, as they not only promote inflammation but also play an inhibitory role. This article outlines the role of B cells in the development of SSc, including the latest research.

## Introduction: role of B cells in autoimmune disease

**Figure 1:**
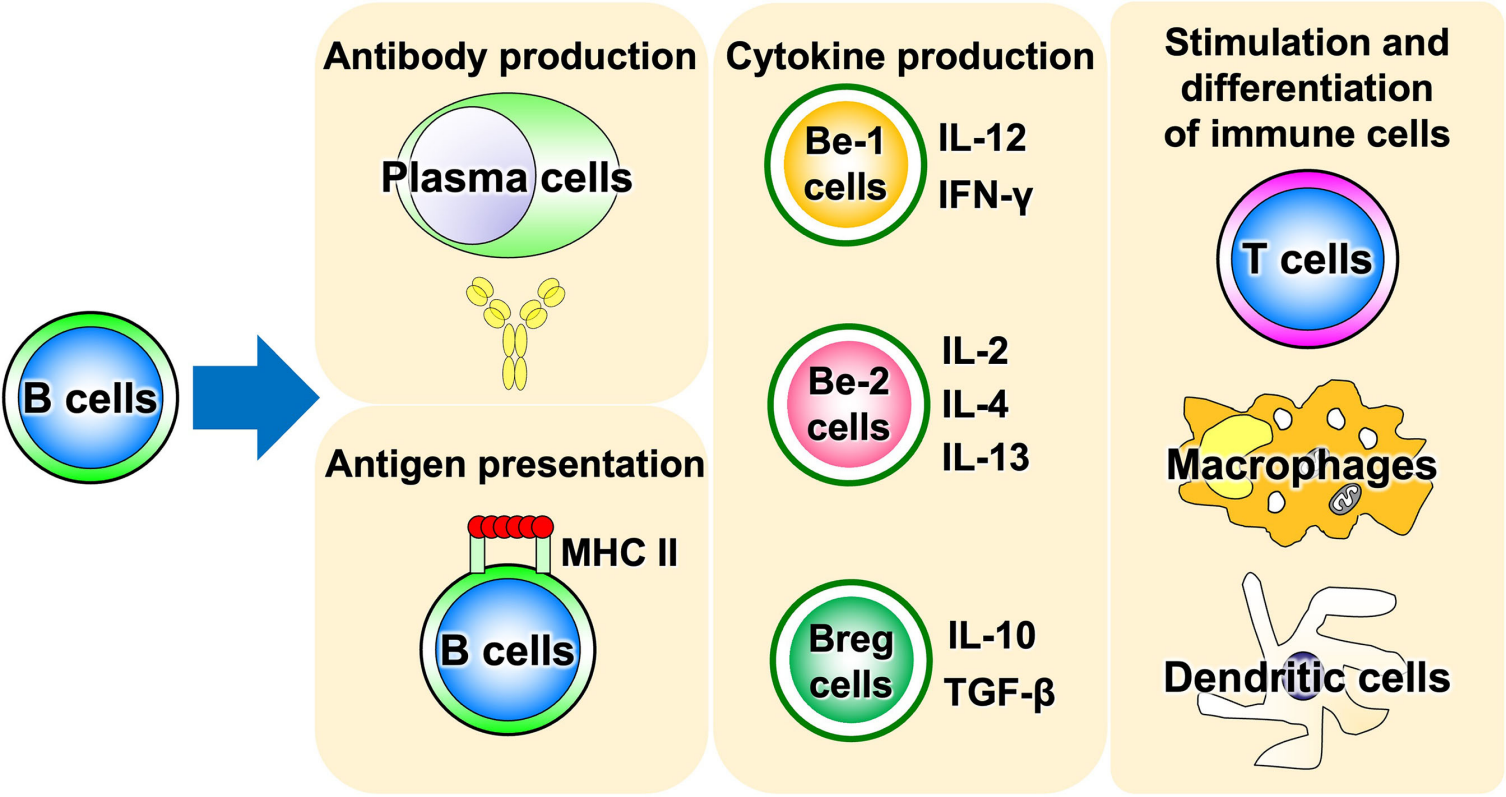
Various functions of B cells. B cells play a central role in the immune system, possessing a variety of functions such as antigen presentation, cytokine production, and induction of differentiation and activation of other immune cells, in addition to antibody production. Be cell, cytokine-producing effector B cell; Breg cell, regulatory B cell; MHC II, MHC class II.

**Figure 2:**
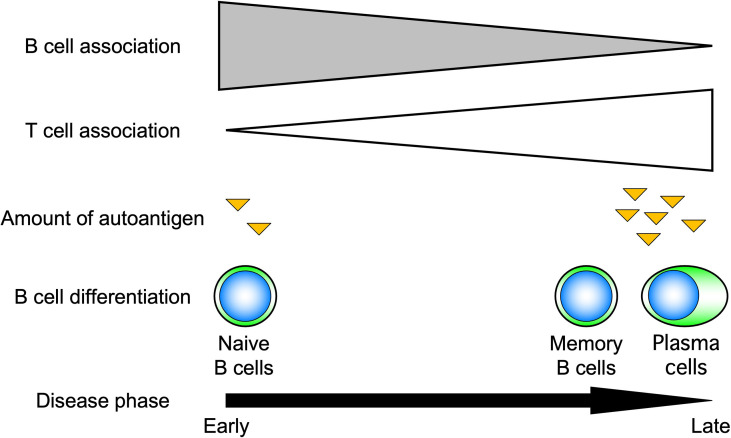
Role of B cells in response to the amount of antigen. B cells induce T-cell activation by efficiently presenting antigens when antigen levels are low. For this reason, B cells are thought to play an important role, especially in the early stages of autoimmune diseases.

Advances in immunology have revealed that B cells play an important role in the immune system by performing a variety of functions in addition to their best-known antibody-producing capacity (**Figure 1**). For example, B cells act as antigen-presenting cells, as do dendritic cells and macrophages (1–3). Antigen-presenting B cells engage in antigen-specific cognate interactions with T cells that recognize peptides presented on the major histocompatibility complex (MHC), during which co-stimulatory molecules such as CD40, CD80, and CD86 are expressed on B cells and transmit signals to T cells. T cells are activated by the simultaneous stimulation of T-cell receptors by specific antigenic peptides and signaling through co-stimulatory molecules. In particular, antigen presentation and co-stimulation of B cells to T cells are important for CD4^+^ T-cell activation when sensitized to small amounts of antigen (**Figure 2**), which is thought to play a major role in the development of autoimmune diseases (4–8). Indeed, in systemic sclerosis (SSc), the importance of CD4^+^ T cells to the pathogenesis has been indicated (9). In addition, B cells regulate lymphoid tissue construction and regeneration, and mice lacking B cells show marked decreases in thymocytes, splenic dendritic cells, and T cells (1–3).

**Figure 3:**
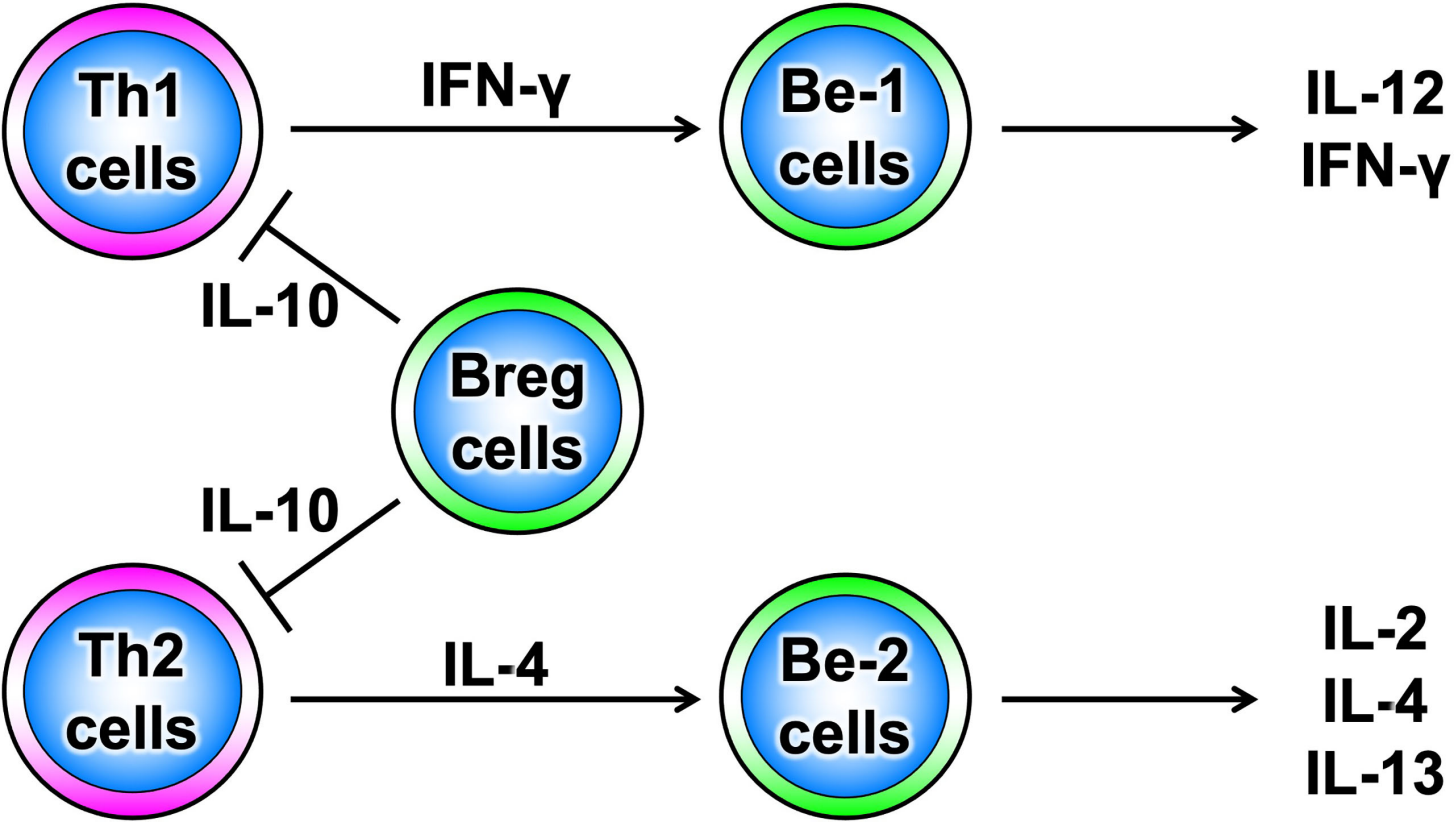
Diversity of B-cell subsets. As with T cells, B cells have subsets that are classified according to the cytokines they produce and are thought to function differently.

**Figure 4:**
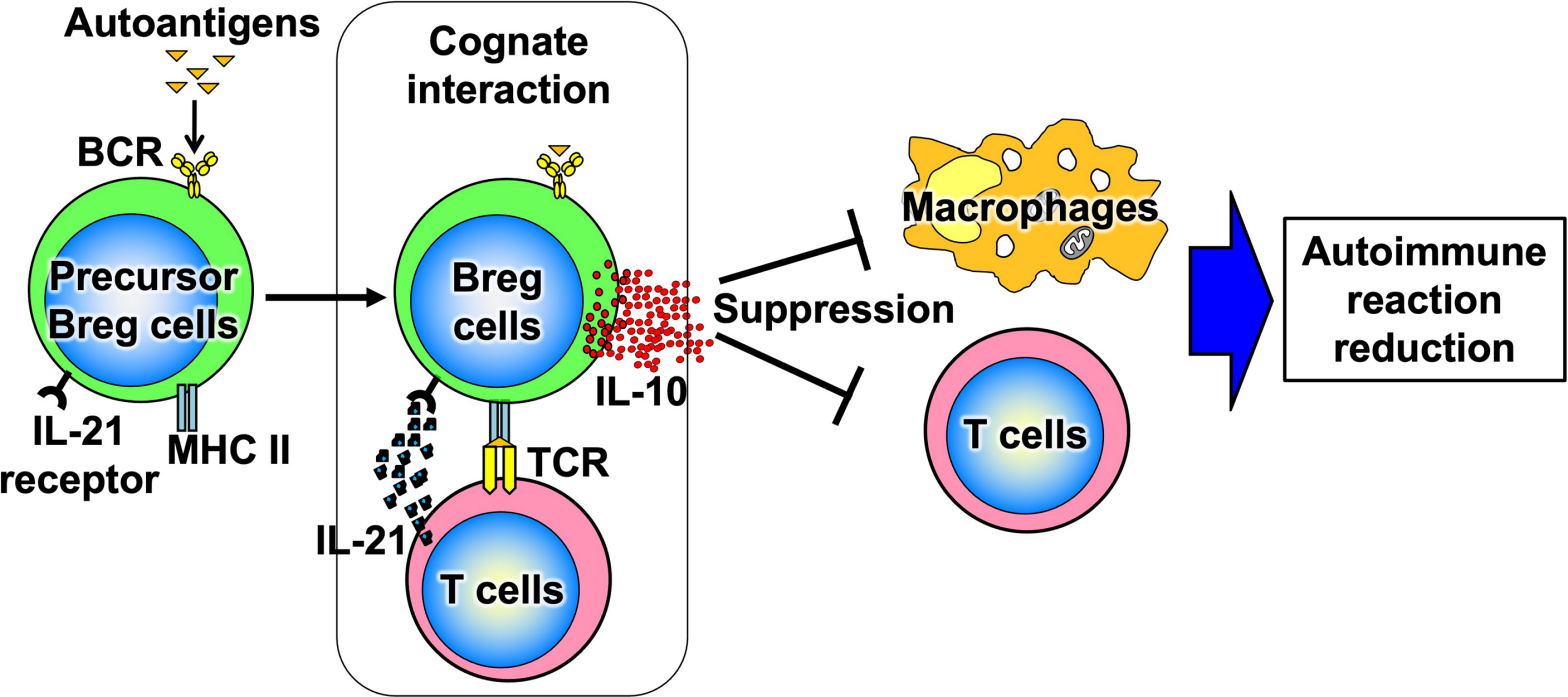
Antigen-specific effects of Breg cells. When Breg cell progenitors encounter autoantigens, they differentiate and produce IL-10. Thus, Breg cells suppress excessive immune responses in an antigen-specific manner.

B cells have recently been shown to produce a variety of cytokines, which, like T cells, can be divided into subsets such as cytokine-producing effector B cells (Be cells) and regulatory B cells (Breg cells), depending on the type of cytokine they produce (**Figure 3**) (10). Be cells are divided into Be-1 cells that produce T helper (Th) 1-type cytokines such as interferon (IFN)-γ and Be-2 cells that produce Th2-type cytokines such as interleukin (IL)-4 and IL-5. Breg cells produce IL-10 and tumor growth factor-β and are thought to be involved in the suppression of inflammation (11–13). Other new types of Be cells, which produce IL-17 to protect from parasitic infections and new Breg cells that produce IL-35, have been identified (14, 15). Thus, the functional classification of B cells according to cytokine production capacity is expected to continue. Among these multiple B-cell subsets, IL-10-producing Breg cells are thought to be important in suppressing autoimmune and inflammatory responses, and studies in mice have shown that IL-10-producing Breg cells suppress excessive immune responses in mouse models of multiple sclerosis, rheumatoid arthritis, and inflammatory bowel disease (13, 16). However, only a few IL-10-producing Breg cells exist *in vivo*, and the mechanism of induction of their differentiation has long been unknown. Recently, it has been shown that IL-21, IL-4, CD40, and B-cell activating factor stimulation are involved in the differentiation and proliferation of IL-10-producing Breg cells (17). Since these factors are produced in IL-21-producing follicular T cells and stromal cells in lymph follicles, lymph follicles are assumed to be one of the sites where regulatory B cells are induced. Furthermore, IL-10-producing Breg cells have been suggested to suppress excessive immune responses in an antigen-specific manner (**Figure 4**) (17). Thus, B cells differentiate into subsets with various functions upon various stimuli and can be significantly involved in inflammatory diseases, including autoimmune diseases, with antigen specificity.

Here, we outline the role of B cells in the pathogenesis of SSc and summarize the efficacy of pan-B-cell elimination therapy in clinical studies. We also address the diverse subsets of B cells involved in autoimmunity and present direct studies of autoreactive B cells to discuss therapeutic strategies that target more specific B cells than are currently available.

## Involvement of B cells in the development of SSc

SSc is a systemic autoimmune disease characterized by fibrosis; vasculopathy of skin, lung, and other tissues; and autoimmune abnormalities as its three main features (3, 18). Recently, various immunological abnormalities, mainly B cells, are thought to be important in the pathogenesis of SSc. Patients with SSc frequently have hypergammaglobulinemia, and various autoantibodies are detected. In addition, these autoantibodies are closely associated with specific disease subtypes and are used as predictors of disease type in clinical practice. For example, anti-topoisomerase I (topo I) antibodies correlate with diffuse cutaneous SSc (dSSc), while anti-centromere antibodies correlate with localized SSc (19). In addition, antibody titers of anti-topo I antibodies are associated with the degree of fibrosis of the skin and lung, renal vessel damage, and elevated antibody titers during the course of the disease which often reflect exacerbation of SSc activity, whereas decreased antibody titers are correlated with improvement in disease activity (20). Furthermore, antiplatelet-derived growth factor receptor antibodies that can be detected in the serum of SSc patients enhance collagen production in healthy human fibroblasts, and the pro-fibrotic effect of these antibodies has been shown to be suppressed by the platelet-derived growth factor receptor blocker, crenolanib (21).

Abnormal B-cell function other than autoantibody production has been described in SSc (3, 19, 22–24). CD19 is a molecule specifically expressed on B cells that lower the activation threshold of B cells and enhances their activation signals, and the expression level of CD19 is increased by about 20% in SSc patients compared with healthy controls (24). On the other hand, the expression of CD20, which is specifically expressed on B cells, did not differ between SSc patients (24). Furthermore, a high frequency of polymorphisms in the CD19 gene promoter region was observed in SSc patients, and the group with this polymorphism showed increased CD19 expression (23). This suggests that enhanced CD19 expression may be involved in the hypergammaglobulinemia often seen in SSc patients.

Tight-skin (Tsk) mice, mutant mice with marked fibrosis of the skin, are widely known as genetic model mice for SSc because they have hypergammaglobulinemia, antinuclear antibodies, and anti-topo I antibodies. Although TSK mice have a duplication of the fibrillin-1 gene, it is unclear how this abnormality is associated with skin sclerosis and autoimmunity (25). However, CD19 expression is not increased in B cells from TsK mice, but tyrosine phosphorylation of CD19 is consistently upregulated (26). At the same time, tyrosine phosphorylation of Vav, a key signaling molecule located downstream of CD19 signaling, is also enhanced in TsK mouse B cells (26). CD19-deficient Tsk mice showed improved skin sclerosis in addition to suppressed autoantibody production (19). Furthermore, when an anti-CD20 antibody was used to remove B cells from the early stage of skin sclerosis, autoantibody production and skin fibrosis were markedly improved (8). Moreover, in a bleomycin-induced SSc mouse model, administration of an anti-CD20 antibody has been shown to improve skin sclerosis and lung fibrosis (27). Since there is currently no sufficiently effective treatment for fibrosis, vasculopathy, and autoimmune abnormality of SSc, B-cell-targeted therapy is expected to be a new therapeutic strategy for SSc, as described in the next section. Moreover, in Japan, an investigator-initiated clinical trial using rituximab, an anti-CD20 antibody agent, showed efficacy in the treatment of SSc skin sclerosis (28), resulting in the regulatory approval of rituximab as a treatment for SSc in September 2021, ahead in the world.

## Effect of anti-CD20 antibody agents on B cells

**Figure 5:**
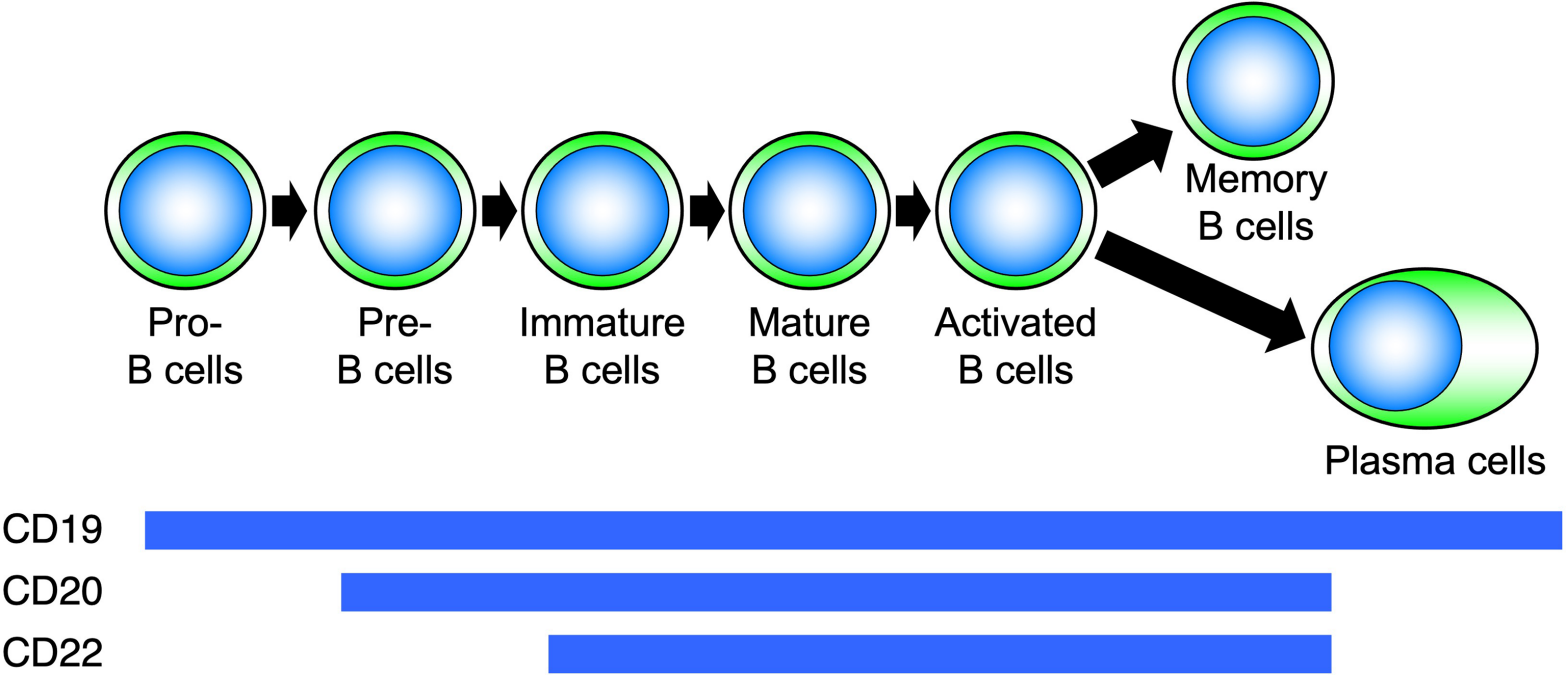
Differentiation stages of B cells and expression of surface molecules. Molecules expressed on B cells at each differentiation stage are different.

CD20 is a four-transmembrane protein with a molecular weight of approximately 33–37 kDa belonging to the MS4A gene family (29). It is a cell membrane molecule that is specifically expressed on B cells and is found from pre-B cells to activated B cells but disappears when B cells differentiate into plasma cells (**Figure 5**). On the other hand, CD19 is expressed on the cell membrane at all differentiation stages after pro-B cell and continues to be expressed after differentiation into plasma cells (30). Hence, B-cell depletion with anti-CD20 antibodies removes pre-B cells to mature B cells, whereas with anti-CD19 antibodies, pro-B cells to plasma cells are removed (31–35). During B-cell activation, CD20 regulates the influx of calcium ions into the cell and cell-cycle progression. Rituximab is a chimeric antibody against CD20, in which the constant region of the IgG-type mouse antibody is replaced by human IgG. Rituximab affects the calcium ion regulation ability of CD20, and the antibody itself directly induces apoptosis by disrupting B-cell signaling and the cell cycle (31–33). In addition, rituximab eliminates B cells through antibody-dependent cell-mediated cytotoxicity (ADCC) and complement-mediated cytotoxicity (CDC) (34). Thus, rituximab induces B-cell elimination through a variety of functions.

Initially, rituximab was developed for the treatment of follicular B-cell lymphoma and mantle cell lymphoma (36). It was further approved for the treatment of other neoplastic diseases, such as B-cell non-Hodgkin’s lymphoma and CD20-positive B-cell lymphoproliferative diseases. It is also used as a treatment for autoimmune diseases, starting with rheumatoid arthritis around 2001, and including granulomatosis with polyangiitis, microscopic polyangiitis, and refractory nephrotic syndrome (37–40). Adverse reactions associated with rituximab administration include transient infusion reactions such as fever, chills, nausea, and allergy-like symptoms, including skin rash and pruritus, which can be controlled with antipyretic analgesics and antihistamines. Unlike those seen with other biologics, rituximab-induced infusion reactions are known to occur more frequently with the first dose and less frequently and less severely with the second and subsequent doses (41). Although the detailed mechanism of rituximab-induced infusion reaction is still unknown, it has been shown that ADCC and CDC induced by rituximab during B-cell depletion increase blood levels of inflammatory cytokines such as tumor necrosis factor-α and IL-6, which are thought to induce an infusion reaction. This hypothesis is supported by the correlation between the concentration of these inflammatory cytokines and the degree of infusion reaction (42). Because rituximab can be administered on an outpatient basis, it may appear to be a safe drug with few problematic adverse events. However, post-marketing surveillance has shown the occurrence of serious infections and progressive multifocal leukoencephalopathy (43). Furthermore, considering the ever-increasing number of diseases for which rituximab is indicated, there is a possibility of unexpected adverse events. Therefore, careful attention should be exercised when administering rituximab.

## B-cell depletion therapy with rituximab in SSc

As mentioned above, rituximab-based B-cell depletion therapy has been investigated in a number of clinical studies for autoimmune diseases. As a result, B-cell depletion therapy with rituximab has been shown to be effective in the treatment of systemic autoimmune diseases such as rheumatoid arthritis, systemic lupus erythematosus, dermatomyositis, and vasculitis (44–48). In SSc, a series of clinical trials have been conducted since around 2009, though with a small number of patients.

Smith et al. reported an open-label study of eight patients with early-stage dSSc within 4 years of onset (49). Rituximab was administered at a dose of 1,000 mg on days 1 and 14, and skin sclerosis was assessed by a modified Rodnan total skin thickness score (MRSS). Using the first dose of rituximab as the baseline, MRSS at 24 weeks showed a significant improvement from a mean of 24.8 points to 14.3 points (p < 0.001). Furthermore, skin biopsy tissue at 12 weeks showed a decrease in collagen fibers and myofibroblasts, as well as the disappearance of B cells infiltrating the skin, which histologically proved the efficacy of rituximab. In contrast, there was no change in SSc-associated interstitial lung disease or HAQ score, which reflects the quality of life of SSc patients.

Lafyatis et al. performed an open-label study with 15 early-stage dSSc cases less than 18 months post-onset using the same dosing regimen as Smith et al. (50). The MRSS evaluation at 24 weeks and 1 year after rituximab treatment showed no improvement in skin sclerosis when compared between pretreatment and 1 year after treatment. However, histological examination showed significant improvement in myofibroblast score and disappearance of B cells infiltrating the skin, suggesting an effect of rituximab on skin fibrosis. This study also did not find a significantly different effect of rituximab on SSc-associated interstitial lung disease but did find a trend toward improvement in percent predicted forced vital capacity (%FVC). They concluded that the reason for the lack of a significant difference was the high variability in the %FVC values obtained in each case.

Daoussis et al. presented an open-label, randomized, controlled trial of 14 dSSc patients (51). Eight and six patients with dSSc matched for MRSS, %FVC, and percent predicted diffusing capacity of the lung for carbon monoxide (%DLco) at baseline were randomly assigned to receive rituximab or placebo, respectively. Rituximab was administered at a dose of 375 mg/m^2^/week per dose for 4 weeks, which was repeated for a total of 2 cycles, one at baseline and the other 24 weeks later. In the rituximab-treated group, the mean %FVC and %DLco showed significant improvement in respiratory function tests, increasing from 68.1% to 75.6% (p = 0.002) and from 52.2% to 62.0% (p = 0.02), respectively, compared to baseline and 1 year later. In the placebo group, the %FVC and %DLco values showed a decreasing trend but no significant worsening over the course of 1 year. However, in the median value study, %FVC and %DLco improved by 10.3% and 19.5%, respectively, in the rituximab group, while they decreased by 5% and 7.5%, respectively, in the placebo group. With regard to skin sclerosis, the rituximab-treated patients showed a mean improvement of 8.4 points from 13.5 points on the MRSS (p = 0.0003), while the placebo group showed an improvement of 9.7 points from 11.5 points (p = 0.16). They continued to observe the patient over the next 2 years with four cycles of dosing and reported the continued efficacy of rituximab (52).

In 2015, the European Scleroderma Trial and Research group (EUSTAR) reported on a 42-center nested case–control study (53). Skin stiffness improved by an average of 24.0% in the rituximab group at 6 months, compared to 7.7% in the control group (p = 0.03). For SSc-associated interstitial lung disease, the study showed a 7.7% decrease in %FVC in the control group at 7 months, compared with a 0.4% improvement in the rituximab group (p = 0.02).

Bosello et al. have reported an open-label study of 20 dSSc cases with an average of 4 years of observation (54), with a dose of 1,000 mg per dose and rituximab administered at baseline and 2 weeks later. The mean MRSS showed significant improvement from 6 months onward (p = 0.001). With regard to SSc-associated interstitial lung disease, the mean %FVC improved from 64.3% to 71.0% at 1 year (p = 0.03) in the six patients with %FVC <80% at baseline but dropped to 65.7% at the last observation. Their further detailed description states that at the last observation, three of the six patients were unchanged, two had improved by more than 10%, and one had worsened by more than 10%, suggesting that this one worsening case could have significantly lowered the mean value. This study was also performed with immunological examinations. B cells, once eliminated by rituximab, recovered between 6 and 12 months, and total serum IgG and IgA levels remained unchanged throughout the course of the study, with a significant decrease in total IgM levels beginning 6 months later (p = 0.002). In addition, there was no significant change in anti-topo I antibody titer throughout the course of the study. Importantly, the investigators found that additional doses of rituximab in patients with flare-ups of skin hardening 12–18 months after the initial rituximab administration resulted in the improvement of symptoms. Although the number of cases may not be large enough to draw complete conclusions, this suggests that the B-cell depletion effect of rituximab is temporary.

Recently, a total of four centers, principally ours, have conducted double-blind, placebo-controlled trials with rituximab for SSc (28). Fifty-six patients with systemic scleroderma entered the study, 28 of whom were assigned to the rituximab group and 26 to the placebo group, both of whom received the study drug (375 mg/m^2^) intravenously for 4 consecutive weeks. This sample size was determined based on calculations from previous open-label studies (55). All patients met the 2013 classification criteria by the American College of Rheumatology/the European Alliance of Associations for Rheumatology, had an MRSS of at least 10 points, and were 20–79 years of age. To exclude the effects of concomitant medications, patients receiving more than 10 mg/day of prednisolone-equivalent steroids or those treated with immunosuppressive or antifibrotic drugs within the past 4 weeks were excluded. For safety reasons, patients with serious complications such as pulmonary hypertension or renal crisis and patients with %FVC below 60% were also excluded from entry as well. Three allocation factors were applied: duration of disease (>/= 6 years), mRSS (>/= 20 points), and presence of SSc-associated interstitial lung disease. The primary endpoint of mRSS change after 24 weeks of study treatment was -6.30 points in the rituximab group and 2.14 points in the placebo group, with a between-group difference of -8.44 points (95% confidence interval: -11.00 to -5.88 points), indicating a significant improvement in skin stiffness in the rituximab group compared with placebo (p < 0.0001). In addition, at 4 weeks after the first dose of the study drug, the rituximab group showed an improvement in MRSS of 1.41 points, and the placebo group showed a worsening of 0.14 points, with the difference between the groups already significant at -1.55 points (95% confidence interval: -2.85 to -0.24 points, p = 0.02). This suggests that the efficacy of rituximab for skin sclerosis in SSc can be expected at a relatively early phase of treatment. Subanalyses on skin sclerosis included duration of disease (>/< 6 years), presence of SSc-associated interstitial lung disease, baseline mRSS (>/< 20), gender, age (median age >/< 48), disease type (dSSc/limited cutaneous type SSc), prior treatment, and serum CRP levels (>/< 0.3 mg/dl), respectively; all analyses showed the efficacy of rituximab for mRSS, indicating that rituximab may be effective against skin sclerosis in a broad group of SSc patients. Furthermore, this trial evaluated %FVC in 48 patients (25 in the rituximab group and 23 in the placebo group) who had SSc-associated interstitial lung disease. At 24 weeks after the first dose of the study drug, %FVC improved by 0.09% in the rituximab group and worsened by 2.87% in the placebo group. The difference between the groups was 2.87% (95% confidence interval: 0.08%–5.84%), with the rituximab group significantly better than the placebo group (p = 0.04). This trial had a high proportion of patients with mild SSc-associated interstitial lung disease, but a subanalysis of only patients with baseline %FVC less than 80% also showed a trend toward better %FVC in the rituximab group than in the placebo group. These results indicate that rituximab may be an effective treatment for SSc-associated interstitial lung disease. However, improvement in SSc-associated interstitial lung disease was seen later than improvement in skin sclerosis. Based on the above, B-cell depletion therapy is considered to be effective for SSc. In fact, in Japan, rituximab was approved by the regulatory authorities in December 2021 for the treatment of SSc that meets the diagnostic criteria.

Thus, a certain amount of data has been accumulated on rituximab, but in recent years, inebilizumab, a humanized anti-CD19 monoclonal antibody, was also approved for the first time in the United States in 2020 for the treatment of neuromyelitis optica spectrum disorder, an autoimmune disease, opening a new avenue for pan-B-cell depletion therapy for autoimmune diseases (35). With regard to SSc, a small, double-blind, placebo-controlled trial of 28 patients was also conducted as a phase I study, in which patients who received inebilizumab showed an average decrease in MRSS of 5.4, compared with an increase of 2.3 points for placebo patients (56). It is hoped that further data will be accumulated in the future to clarify the usefulness of the anti-CD19 antibody therapy.

## B cells with variable functions and diverse subpopulations

As mentioned above, B-cell depletion therapy is one of the effective treatments for SSc. However, eliminating all B cells remains a difficult task, since B cells are an important component of the body that plays a role in infection defense. In this section, we will focus on the various functions and diverse subpopulations of B cells and discuss the pathogenic potential of B cells.

**Figure 6:**
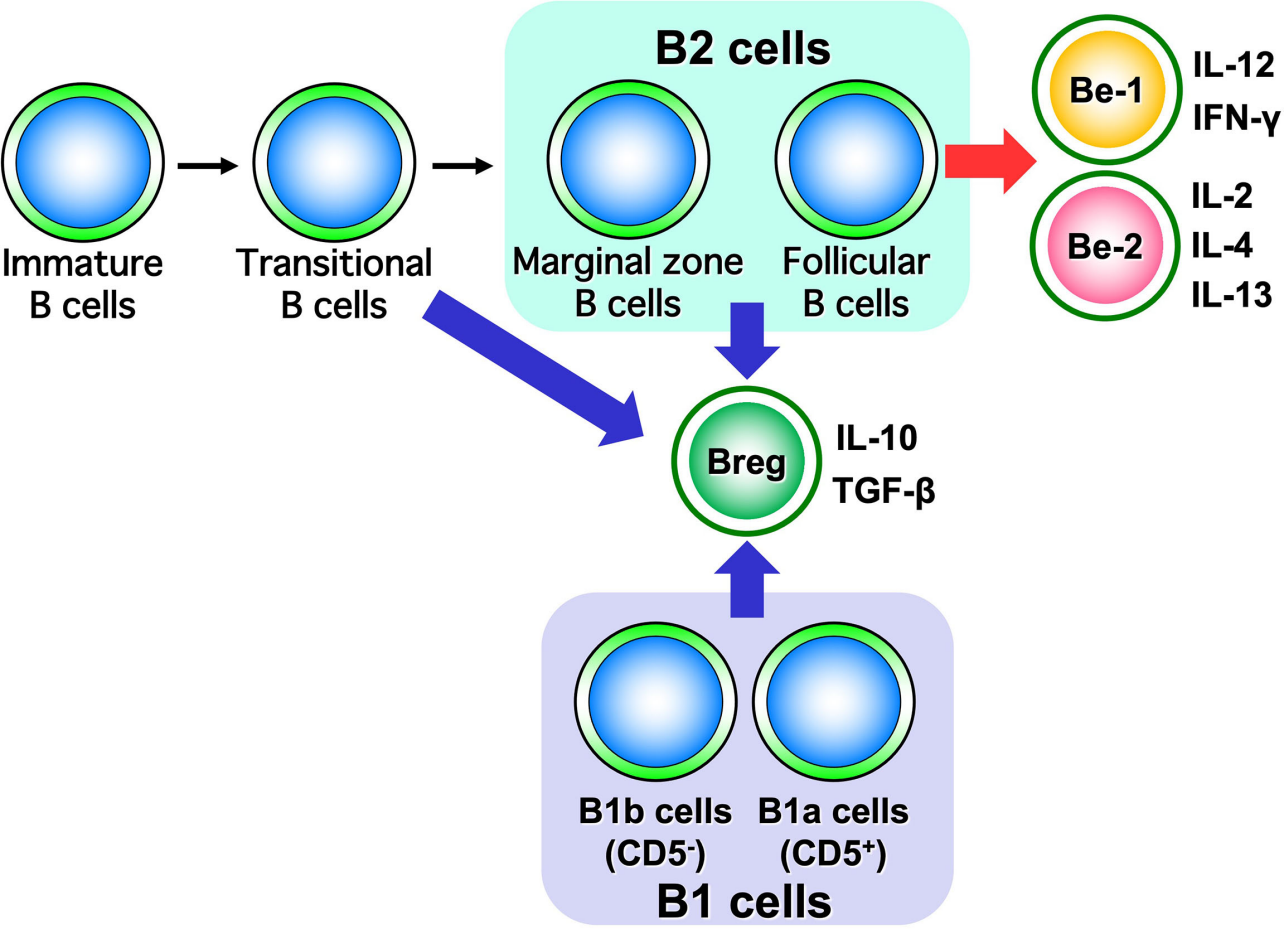
Classification of B cells by cytokine-producing capacity. B cells are classified into Be cells and Breg cells based on the type of cytokines they produce. It has been suggested that Be cells are part of B2 cells, while Breg cells are part of both B1 and B2 cells.

Advances in immunology have revealed that B cells have a wide variety of functions (57). Traditionally, B cells were thought to have only a role in producing antibodies, but it is now suggested that they play a central role in the immune system by presenting antigens and promoting the activation and differentiation of other immune responsible cells (**Figure 1**). Thus, B cells are also recognized as important in autoimmune diseases, where an excessive immune response is thought to play a major role in their pathogenesis. Self-antigen stimulation *via* the B-cell receptor (BCR) induces activation of autoreactive B cells, which in turn may play a major role in the formation and progression of autoimmune diseases. It has become clear that B cells, like T cells and macrophages, produce a wide variety of cytokines (10). The concept of classifying B cells according to the type of cytokines that they produce has been attracting attention, and B cells which produce anti-inflammatory cytokines have been designated as Be cells, while B cells which produce anti-inflammatory cytokines have been labeled as Breg cells (**Figure 6**).

### Be cells and autoimmune diseases

Be cells, referred to here in the sense of having the function of producing cytokines, are classified into Be-1 cells and Be-2 cells based on the type of cytokines they produce (**Figure 3**) (10). Be-1 cells produce Th1-type cytokines such as IFN-γ and IL-12 upon stimulation by Th1 cells or Th1-type antigens such as *toxoplasma gondii* and *Borrelia burgdorferi* (58, 59). Be-1 cells were identified in an infection experiment that induced a Th1-type response in mice (58). Be-2 cells, on the other hand, were identified in a mouse model of parasitic infection; Be-2 cells have been shown to produce Th2-type cytokines, including IL-2, IL-4, and IL-12, upon stimulation by Th2 cells, Th2-type antigen stimulation such as *Heligmosomoides polygyrus*, and allergic responses (58, 60). Moreover, B cells are classified into B1 cells and B2 cells based on their origin and distribution. B1 cells originate in the liver during embryonic development and are distributed in mucosal tissues, the abdominal cavity, and the thoracic cavity. B2 cells originate in the bone marrow and migrate to lymphoid tissues. B2 cells are divided into marginal zone B cells and follicular B cells based on their distribution in lymphoid tissues. Marginal zone B cells are important for innate immunity, while follicular B cells are important for antigen-specific acquired immunities. Be cells have a follicular B-cell phenotype, suggesting that Be cells may be deeply involved in antigen-specific responses in autoimmune diseases (61).

### Breg cells and autoimmune diseases

By producing suppressive cytokines such as IL-10 and transforming growth factor-β, Breg cells are thought to exert immunosuppressive functions (**Figure 3**) (62). Unlike Be cells, which are localized to follicular B cells, Breg cells have phenotypic features such as CD5-positive B1a cells, transitional B cells, follicular B cells, and marginal zone B cells (11, 17, 63–65). Several studies in mice and humans have suggested that the role of Breg cells differs depending on which fraction they belong to (11, 17, 63–65). These studies have shown that in mice, Breg cells in the B1a fraction suppress excessive immune responses to innate immunity during the neonatal period, while Breg cells in the B2-cell, marginal zone B-cell, or transitional B-cell fraction have been found to suppress autoimmune responses in the adult period (63, 64). In humans, Breg cells with marginal zone B-cell traits that strongly express CD1d or transitional B cells that express both CD24 and CD38 are known to be involved in the pathogenesis of many autoimmune diseases (65). The adoptive transfer of Breg cells into mouse models of autoimmune diseases markedly suppresses autoimmune reactions and associated symptoms and has attracted attention as a therapeutic approach for human patients (17).

## Direct investigation of autoreactive B cells

As mentioned above, the involvement of autoreactive B cells in autoimmune diseases is considered to be significant. However, since only a few autoantigen-reactive B cells exist *in vivo*, a direct analysis, for example, single-cell analysis at the protein level, is technically difficult and has not yet been fully performed (64). Thus, the function of autoantigen-reactive B cells remains a black box, the same as the pathogenicity of many autoantibodies. Nevertheless, the importance of B cells in autoimmune diseases is evident from the fact that B-cell elimination therapy is useful in many autoimmune diseases, such as vasculitis, rheumatoid arthritis, systemic lupus erythematosus, and SSc (66). The therapeutic effect of B-cell depletion therapy on these diseases is not necessarily exerted by lowering the antibody titer of autoantibodies, which strongly suggests that B cells themselves affect autoimmune diseases in an antibody-producing capacity-independent manner. In other words, it is assumed as a mechanism that B cells influence the function of other cells responsible for immunity, including T cells, through their ability to produce cytokines (67). Furthermore, a retrospective analysis of data obtained from our investigator-initiated clinical trial with rituximab for SSc showed that rituximab was indeed more effective in patients with higher B-cell counts in the peripheral blood at baseline, while the B-cell depletion therapy was less effective in those with lower B-cell counts (68). Here, we describe a direct examination of autoreactive B cells that has been conducted recently.

### Study on autoreactive B cells using the Breg cell proliferation system

We have discovered factors involved in the proliferation and differentiation of Breg cells and established a system for their proliferation from single cells *in vitro* (17). Analysis using this system showed that the immunosuppressive effects of Breg cells are dependent on antigen-specific B-cell and T-cell interactions, the so-called cognate interaction, mediated by MHC class II (MHC II) and T-cell receptors (TCRs) (**Figure 4**). It has been suggested that IL-21 may be a potent inducer of Breg cells in this proliferative system; IL-21 is produced by follicular helper T cells, plays a central role in the formation of lymphatic follicles *in vivo*, and is a critical factor essential for antigen-specific inflammatory responses (69, 70). Thus, it is suggested that B cells may be involved in autoantigen-specific inflammatory responses in autoimmune diseases *via* IL-21 during antigen-specific responses in lymph follicles but also may function as a brake to suppress excessive immune responses by differentiating into regulatory B cells. Indeed, we have shown that antigen-specific Breg cells from the mice immunized with myelin-related proteins suppress inflammatory responses more potently than Breg cells from non-immunized mice in a combined study of an experimental autoimmune encephalomyelitis model and the Breg-cell proliferation system (17). This implies that Breg cells exert their anti-inflammatory effects in an antigen-specific manner.

### Investigation of single autoreactive B cells at the protein level using ultra-sensitive analytical methods

**Figure 7:**
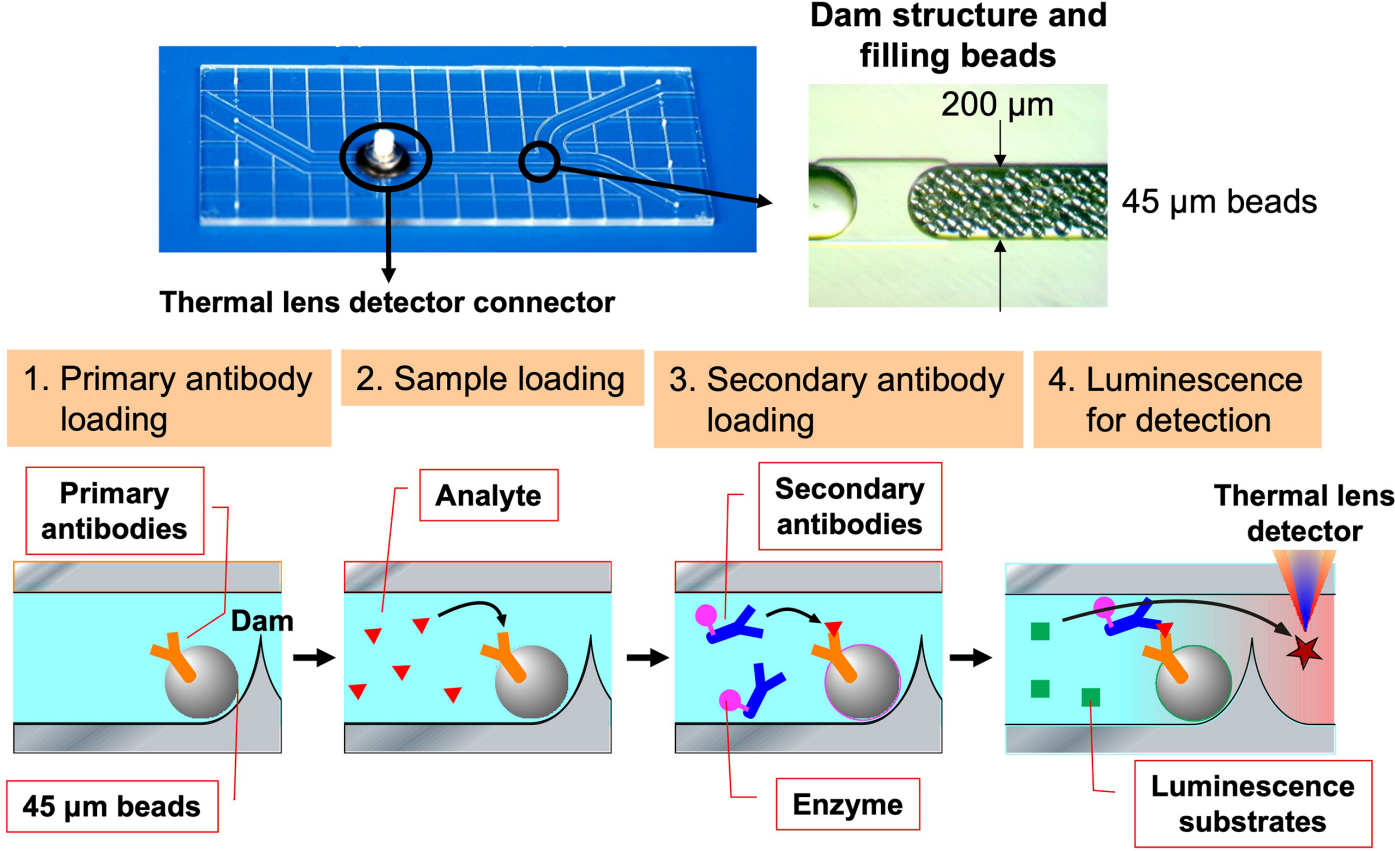
The device enables measurement of cytokine levels produced by a single B cell. The ultra-sensitive protein analysis device was developed through a medical-engineering collaboration between the University of Tokyo Graduate School of Engineering and Medicine, which uses a 3 cm × 7 cm glass substrate to create a flow channel for bead enzyme-linked immunosorbent assay to measure cytokine levels with 100 to 1,000 times greater sensitivity than conventional methods. Polystyrene beads attached to primary antibodies are filled into this device and restrained by a dam formed in the flow channel. The analyte in the sample is captured by the primary antibody and subsequently labeled by the second antibody combined with the enzyme. Finally, the enzymatic reaction causes the substrate to emit light, and the signal is detected by thermal lens microscopy.

**Figure 8:**
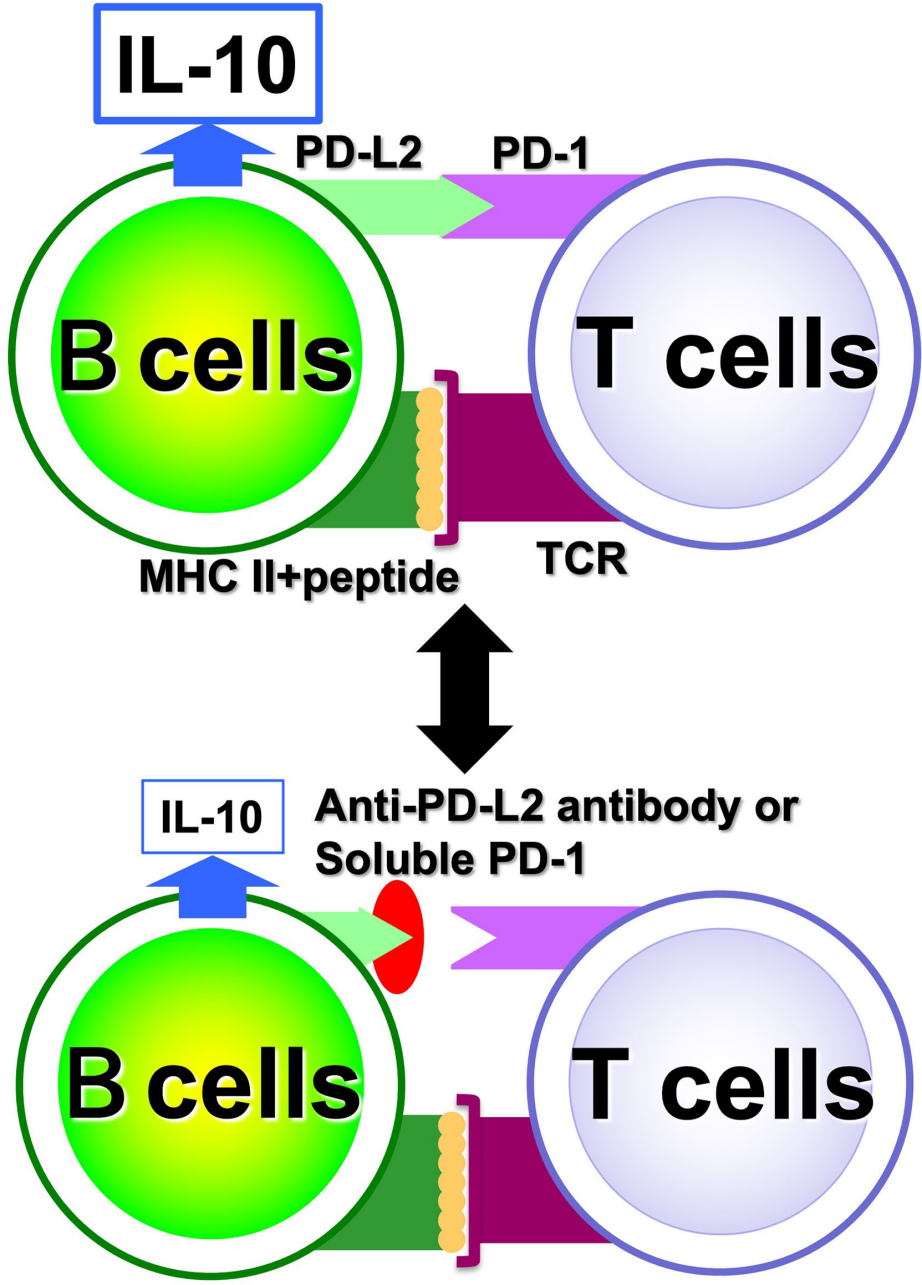
Regulation of antigen-specific responses of B cells by PD-1. In antigen-specific interactions between B cells and T cells, PD-1 and PD-L2 binding is involved in B cell IL-10 production; when PD-1 and PD-L2 binding is inhibited, IL-10 released from B cells is reduced.

With regard to Breg cells, the aforementioned proliferation system has allowed us to examine their self-reactivity in a more direct manner. However, the autoreactivity of Be cells has not yet been studied directly enough due to the small number of autoreactive B cells present *in vivo*. Hence, through medical-engineering collaboration research, we have developed the device shown in **Figure 7**, which enables measurement of trace amounts of cytokines produced by a small number of autoreactive B cells (71–74). This device is fabricated by surface modification technology of glass substrates and low-temperature bonding technology (71, 72). By using fluid control technology, it is possible to perform ELISA using polystyrene beads with capture antibodies, which are filled into μm units of microfluidic channels. Furthermore, the fluorescent signal is detected with high sensitivity by a measurement system using a thermal lens microscope, making it possible to measure trace amounts of cytokines produced by B cells with 100- to 1,000-fold higher sensitivity than conventional plate ELISA (73, 74). Using this device, we examined B cells at the single-cell level in a topo I-induced SSc mouse model induced by immunization with topo I antigen, a disease-specific autoantigen for SSc, that we established ourselves (75). The Topo I-induced SSc mouse model closely mimics SSc with anti-topo I antibodies in the blood and fibrosis in the skin and lungs, as in human dSSc patients (75). In this topo I-induced SSc mouse model, programmed death (PD)-1 and one of its ligands, PD-L2, play an important role in the interaction between T cells and B cells, and inhibition of their binding suppressed IL-10 production from topo I antigen-specific B cells and enhanced autoimmune responses (**Figure 8**) (76). Breg cells that secrete IL-10 are known to exert antigen-specific inhibitory functions through the interaction between MHC II expressed on themselves and the TCR of T cells, as described earlier (17). In addition, PD-1 may similarly regulate the immune system not only by non-specifically conveying inhibitory signals to T cells but also by affecting antigen-specific T cell–B cell interactions. Furthermore, topo I-reactive B cells from topo I-induced SSc model mice produced inflammatory suppression cytokines such as IL-10 and IL-35 when the affinity of BCR for topo I antigen was low, while they produced inflammatory cytokines such as IL-6 and IL-23 when the antigen affinity for topo I antigen increased with the frequency of topo I antigen-induced autoimmune triggers (71). Similarly, in SSc patients positive for anti-topo I antibodies, B cells with low BCR affinity for topo I antigen produced IL-10 and IL-35, whereas B cells with high antigen affinity for topo I antigen produced IL-6 and IL-23 (71). Furthermore, these high-affinity topo I-reactive B cells were shown to differentiate T cells into IL-17-producing Th17 cells *via* IL-6 and IL-23. In SSc, Th17 cells have been shown to induce fibroblasts and vascular endothelial cells to produce collagen and inflammatory cytokines *via* IL-17A and IL-17F, as well as promote B-cell proliferation, differentiation, and activation (77–79). These findings suggest that the increased antigen affinity of B cells for autoantibodies is involved in the production of proinflammatory cytokines from B cells and influences the pathogenesis of SSc.

## Concluding remarks and future

As described above, it is strongly suggested that B cells, which are producers of autoantibodies closely related to the disease type, play a major role in the pathogenesis of SSc. The importance of B cells in SSc is also supported with the effectiveness of B-cell depletion therapy. Although autoreactive B cells are considered to be particularly important, their presence *in vivo* is minimal, which has made their investigation difficult. Therefore, to discuss the function of autoantigen-specific autoreactive B cells, single-cell analysis at the protein level is required, allowing analysis of the cytokines produced. Single B-cell analysis at the protein level has not been fully performed in the past, but due to advances in technology, it is gradually being conducted in recent years, as summarized in this article. It is hoped that further research will more clearly characterize autoreactive B cells than today and develop a safe, specific treatment with fewer side effects that will replace the current pan B-cell depletion therapy.

## Author contributions

Conceptualization: AY, SS; Supervision: AY; Writing - original draft: AY; Writing - review and editing: AY, TF, ES, AY-O, SS. All authors contributed to the article and approved the submiitted version.

## Acknowledgments

I would like to express my sincere gratitude toward my supervisor, SS, for his contribution and professional help to this review.

## Conflict of interest

The authors declare that the research was conducted in the absence of any commercial or financial relationships that could be construed as a potential conflict of interest.

## Publisher’s note

All claims expressed in this article are solely those of the authors and do not necessarily represent those of their affiliated organizations, or those of the publisher, the editors and the reviewers. Any product that may be evaluated in this article, or claim that may be made by its manufacturer, is not guaranteed or endorsed by the publisher.
